# Letter from Dr. N. S. Jenkins, Dresden

**Published:** 1899-01

**Authors:** N. S. Jenkins

**Affiliations:** Dresden


### Foreign Correspondence.

### LETTER FROM DR. N. S. JENKINS, DRESDEN.

DRESDEN, November 30, 1898.

TO THE EDITOR:

SIR,—In the October number of the *Dental Practitioner and Advertiser*, edited by Dr. Barrett, of Buffalo, appears the extraordinary announcement that "Dr. Norman W. Kingsley, formerly of New York, has located in Dresden, Germany, as the assistant of Dr. Jenkins. He has charge of the laboratory and prosthetic work."

The only possible basis of such a ridiculous statement is that, when upon a friendly visit at my country house, a few years ago, Dr. Kingsley, during the illness of one of my partners, although at that time far from well himself, kindly came into town and gave me, in some special cases, the inestimable advantage of his great skill and wide experience.

As the publication of the *Dental Practitioner and Advertiser* has been suspended with the October number, I shall be most grateful if you will kindly publish this statement in your esteemed journal, to correct a rumor which is as unjust to my distinguished friend as it is grotesquely flattering to me.

I am, sir,

Yours very faithfully,

[Signed] N. S. JENKINS.

[The above communication is given insertion in justice to the parties interested. It is proper to say, also, in justice to the editor of the *Dental Practitioner and Advertiser*, Dr. Barrett, that the writer had repeatedly heard the matter stated as a fact long before it was published in that journal.—ED.]

